# Biological and Histopathological Investigations of Moclobemide on Injured Ovarian Tissue Following Induction of Ischemia-Reperfusion in Rats

**Published:** 2012-06-19

**Authors:** Metin Ingec, Muhammet Calik, Cemal Gundogdu, Ali Kurt, Mehmet Yilmaz, Unal Isaoglu, Suleyman Salman, Fatih Akcay, Halis Suleyman

**Affiliations:** 1. Department of Obstetrics and Gynecology, Medical Faculty, Ataturk University, Erzurum, Turkey; 2. Department of Pathology, Medical Faculty, Ataturk University, Erzurum, Turkey; 3. Department of Pathology, Education and Research Hospital, Erzurum, Turkey; 4. Nene Hatun Obstetrics and Gynecology Hospital, Erzurum, Turkey; 5. Obstetrics and Gynecology Hospital, Igdýr, Turkey; 6. Department of Biochemistry, Medical Faculty, Ataturk University, Erzurum, Turkey; 7. Department of Pharmacology, Medical Faculty, Ataturk University, Erzurum, Turkey

**Keywords:** Moclobemide, Ischemia/Reperfusion, Oxidant/Antioxidant, Rat

## Abstract

**Background:**

The effects of moclobemide on damaged ovarian tissue induced by ischemia- reperfusion and damaged contralateral ovarian tissue were investigated in rats, biochemically and histologically.

**Materials and Methods:**

In this experimental study, 40 rats were equally divided into four groups: 10 mg/kg moclobemide, 20 mg/kg moclobemide, ischemia/reperfusion control, and intact control groups. A 2-2.5-cm-long vertical incision was made in the lower abdomen of each rat in order to reach the ovaries, after which a vascular clip was placed on the lower side of the right ovary of each animal in the two treatment groups and the ischemia-reperfusion control group, but not in the healthy (intact control) animal group. The purpose of this procedure was to create ischemia over the course of three hours, then the clips were unclamped to provide reperfusion for the next two hours. At the end of the two hours of reperfusion, all the animals were killed by high-dose anaesthesia and their ovaries were taken and subjected to histological and biochemical (malondialdehyde, nitric oxide, glutathione) studies.

**Results:**

The obtained results showed that moclobemide suppressed nitric oxide and malondialdehyde production in the ischemia-reperfusion damage area, and prevented the decrease in endogenous antioxidant levels (glutathione) in the rat ovarian tissue. Moclobemide also prevented infiltration of leukocytes to the ovarian tissue. These results showed that moclobemide protected ovarian tissue against ischemiareperfusion injury.

**Conclusion:**

This study shows that moclobemide represses malondialdehyde and nitric oxide production in the rat ovarian tissue subjected to ischemia-reperfusion injury and keeps the endogenous antioxidant glutathione level from decreasing. Moclobemide also inhibits leukocytic migration into ovarian tissue following ischemia-reperfusion injury. From these results, it is suggested that moclobemide can be used in the treatment of ovarian ischemia-reperfusion injury.

## Introduction

Ischemia is defined as a lack of oxygen in tissues or organs resulting from reduction in arterial or venous blood flow or insufficient perfusion. It causes accumulation of toxic metabolites in tissues or organs, leading to cell death (1). Reperfusion is needed to protect tissues from toxic metabolites. It can, however, initiate a reactive process, causing even more injury to the ischemic tissue (2). Reperfusion injury is produced by a complex mechanism involving reactive oxygen species (ROS), endothelial factors (such as nitric oxide of endothelial origin), and neutrophils (3, 4). ROS have been called the mediators of the reperfusion aspect of ischemia-reperfusion (I/R) injury.

Membrane lipids are among the cellular structures that are most sensitive to injury from free radicals (5), which cause lipid peroxidation. Malondialdehyde (MDA), the end product of lipid peroxidation, is widely used as an index of oxidative status (6). The irreversibility of cell membrane damage due to lipid peroxidation is well known (7). The role of nitric oxide (NO) of endothelial origin is also widely recognised: NO can be released into the circulation in response to stress due to hypoxia or mechanical injury (8). Leukocyte activation is also observed during I/R injury, which releases ROS from activated white blood cells (WBC) (9-11). Antioxidant defence mechanisms, however, are generated in the body against ROS (12).

Moclobemide, the drug used in our study, is an antidepressant drug and a selective inhibitor of the monoamine oxidase-A (MAO-A) (13). It has fewer secondary effects than other classic MAO inhibitors. Antidepressant drugs are also used in the treatment of multiple non-psychiatric diseases (14), and it has been experimentally demonstrated that moclobemide increases melatonin synthesis in animals (15). Further, melatonin is known to have a potent antioxidant effect (16). Thus, it can be inferred that moclobemide could have an antioxidant effect via an indirect mechanism. In the literature review, we could not find any paper investigating the effect of moclobemide on ovarian I/R damage or its direct antioxidant activity. The objective of our study is; therefore, to investigate the effect of moclobemide on certain indicators of oxidation-antioxidation, and evaluate this effect by histological examination.

## Materials and Methods

### Animals

This experimental study involved a total of 40 albino Wistar female rats weighing between 200 and 215 grams, provided by Ataturk University Medical Experimental Application and Research Centre. The animals were fed and kept at the room temperature (22˚C) in groups. The Rats were divided into the following four groups (10 rats per group): moclobemide (10 mg/kg) + I/R group, moclobemide (20 mg/kg) + I/R group, I/R group, and intact control group without moclobemide treatment. The experiments were performed in accordance with the national guidelines for the use and care of laboratory animals and were approved by the Local Animal Care Committee of Ataturk University.

### Chemicals

Of the chemicals used in the study, thiopental sodium was procured from Sigma Co (Munich, Germany) and moclobemide from Deva Drugs (Istanbul, Turkey).

### Experimental method

Surgical interventions were performed on the rats in sterile conditions and adequate laboratory environment under intraperitoneal (i.p.) anaesthesia by thiopental sodium (25 mg/kg). Four groups of ten animals were formed: moclobemide groups 1 and 2 were received 10 and 20 mg/kg moclobemide by oral gavage, respectively; the I/R control group and the intact control group were given the vehicle, i.e., distilled water, in the same volume and by the same route as the treatment groups. After thiopental injection, the rats were left for the appropriate time for surgery. When the animals remained motionless on their backs, the surgery was proceeded. At this time, a 2-2.5 cm long vertical incision was made in the lower abdomen of each animal to penetrate the ovaries, then placed a vascular clip in the lower side of the right ovary (in the utero-ovarian contact area) of the rats in the two treatment groups and control group, but not in the healthy animal group. Ischemia took place over three hours, after which the clips were unclamped to provide reperfusion for the next two hours. At the end of the two hours of reperfusion, all rats were killed by high-dose anaesthesia, and their right and left ovaries were taken and subjected to histological and biochemical studies. Observations made about the animals in the treatment groups were compared to those in the I/R control and intact control groups.

### Statistical analyses

All data were subjected to one-way analysis of variance (ANOVA) using Statistical Package for the Social Sciences (SPSS) 18.0 software. The differences among groups were attained using the least significant difference option, and significance was declared at p<0.05. Results were given as mean ± standard deviation.

### Biochemical analysis of ovarian tissue

A sample included both damaged and healthy area of the tissue from the collected ovary was weighed (weight 0.2 mg). The samples were homogenized in ice with 2-ml buffers (consisting of 1.15% potassium chloride solution for malondialdehyde analysis with pH=7.5 and phosphate buffer for the other analyses). Then, they were centrifuged at 4˚C, 10000 rpm for 15 minutes. The supernatant part was used as the analysis sample (17).

### Malondialdehyde analysis

The concentrations of ovarian lipid peroxidation were determined by estimating MDA using the thiobarbituric acid test (18). The rat ovaries were rinsed with cold saline, and the corpus mucosa was scraped, weighed, and homogenized in 10 ml of 100 g/l KCl (Potassium chloride). The homogenate (0.5 ml) was added to a solution containing 0.2 ml of 80 g/l sodium lauryl sulfate, 1.5 ml of 200 g/l acetic acid, 1.5 ml of 8 g/l 2-thiobarbiturate, and 0.3 ml distilled water. The mixture was incubated at 98˚C for 1 hour. Upon cooling, 5 ml of n-butanol: pyridine (15: l) was added. The mixture was vortexed for 1 minute and centrifuged for 30 minutes at 4000 rpm. The absorbance of the supernatant was measured at 532 nm. The standard curve was obtained using 1, 1, 3, 3- tetramethoxypropane (19).

### Nitric oxide analysis

Nitric oxide levels were measured by the Griess reaction (20, 21). Nitric oxide measurement is difficult to achieve because of the chemical short half-life. Nitrate and nitrite levels, which are stable end products of nitric oxide metabolism, were; therefore, used. The measurement mixture [100 μl sample, 100 μl nicotinamide adenine dinucleotide phosphate (NADPH) (50 μmol/l), 100 μl flavin adenine dinucleotide (FAD) (5μmol/l), 20 μl nitrate reductase (200 U/l)] was prepared and incubated for 20 minutes at 37˚C. After this, 25 μl ZnSO4 (300 g/l) was added to this mixture, and in this manner, deproteinization occurred. This mixture was centrifuged for 15 minutes at 1000 rpm. The supernatant part was used as measurement assay. One hundred μl Griess reagent and 100 μl of metaphosphoric acid were added to the supernatant, and a deep purple azo compound was obtained. The Griess reagent consists of 0.5 g sulfanilamide, 12.5 g phosphoric acid, and 0.05 g N-(1-napthyl)- ethylenediamine in 500 ml distilled water. The method is based on a two-step process. In the first step, nitrate is converted into nitrite by nitrate reductase. In the second step, nitrite reacts with the Griess reagent, and at the end of this reaction, a deep purple azo compound is appeared. The absorbance of this deep purple azo compound was measured at 540 nm wavelength by photometric measurement. This azo chromophore accurately determines nitrite concentration as a marker of NOx (17).

### Glutathione analysis

The amount of Glutathione (GSH) in the total homogenate was measured according to the method of Sedlak and Lindsay, with some modifications (22). The sample was weighed and homogenized in 2 ml of 50 mM Tris-HCl buffer containing 20 mM ethylenediaminetetraacetic acid (EDTA) and 0.2 mM sucrose at pH=7.5. The homogenate was immediately precipitated with 0.1 ml of 25% trichloroacetic acid. The precipitate was removed after centrifugation at 4200 rpm for 40 minutes at 4˚C, and the supernatant was used to determine GSH level. One thousand five hundred μl of measurement buffer (200 mM Tris-HCl buffer containing 0.2 mM EDTA at pH=7.5), 500 μl supernatant, 100 μl 5,5-dithiobis (2-nitrobenzoic acid) (DTNB) (10 mM), and 7900 μl methanol were added to a tube, vortexed, and incubated for 30 min at 37˚C. DTNB was used as a chromogen, and it formed a yellowtcoloured complex with SH groups. The absorbance was measured at 412 nm using a spectrophotometer. The standard curve was obtained using reduced glutathione (17).

### Histological examination

After the operations, the ovaries were fixed in a 10% neutral buffered formalin solution, and then embedded in paraffin. The serial sections were cut with a microtome at a thickness of 4 μm and stained with haematoxylene-eosin. The histologic sections were examined for the presence of interstitial edema, vascular dilatation, haemorrhage, and polymorphonuclear neutrophilic infiltrations using an Olympus BX-50 microscope, and photographed. The slides were coded, and semiquantative analysis of the ovarian sections was performed without any knowledge of treatment protocol. The observed changes were graded as follows: grade 0, normal; grade I, mild edema, mild vascular congestion, no haemorrhage, and no leukocytic infiltration; grade II, moderate edema, moderate vascular congestion, no haemorrhage, and no leukocytic infiltration; grade III, severe edema, severe vascular congestion, minimal haemorrhage, and minimal leukocytic infiltration; grade IV, severe edema, severe vascular congestion, haemorrhage, and leukocytic infiltration.

## Results

### Biochemical results

The levels of MDA, NOx, and GSH in the ovarian tissues as well as contralateral ovarian tissues in the four experimental groups are separately shown and summarized in table 1, which shows the mean values, standard deviations, and p-values of the comparison with the corresponding level in the control group.

### Histological results

**Fig 1:**
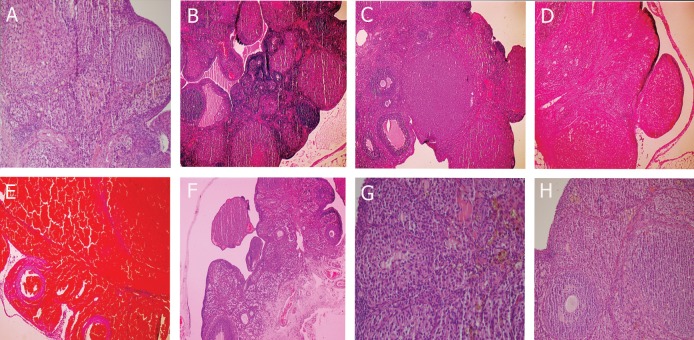
A.The histo-pathological examination of the ovarian tissue of intact control group (Grade 0). B. The histo-pathological examination of the ovarian tissue of Moclobemide 10 mg/kg group (Grade 2). C. The histo-pathological examination of the ovarian tissue of Moclobemide 20 mg/kg group (Grade 1). D . The histo-pathological examination of the ovarian tissue of I/R control group. (Grade 3). E . The histo-pathological examination of the ovarian tissue of I/R control group. (Grade 4). F. The histo-pathological examination of the contralateral ovarian tissue of I/R group (Grade 1). G. The histo-pathological examination of the contralateral ovarian tissue of Moclobemide 10 mg/kg group (Grade 0). H. The histo-pathological examination of the contralateral ovarian tissue of Moclobemide 20 mg/kg group (Grade 0)

The ovarian tissue of the animals in the intact control group was accepted as being normal; the injury level was estimated as grade 0 (Fig 1A). In the group of rats received a moclobemide dose of 10 mg/kg, the injury level was estimated as grade II (Fig 1B), while the injury level was grade I in the moclobemide 20 mg/kg group (Fig 1C). Histological pictures revealed I/R injury was grade III in the four rats (Fig 1D) and was estimated to be grade IV in the remaining six rats (Fig 1E). While in contralateral ovarian tissue of IR control group, the damage severity was determined as grade I (Fig 1F), in contralateral ovarian tissue of 10 and 20 mg/kg moclobemide rat groups, the severity for both was grade 0 (Fig 1G, H).

**Table 1:** Comparing MDA, NOx and GSH levels in different dose of Moclobemide (10 mg/kg and 20 mg/kg) and different experimental groups (Intact control, I/R (Ischemia/Reperfusion) group and contralateral group)


	N	MDA (µmol/g protein)	NOx (µmol/g)	GSH (µmol/g protein)

**Moclobemide (10 mg/kg)**	10	4.6± 0.36**	10.6 ± 0.23*	2.5± 0.24**
**Moclobemide (20 mg/kg)**	10	4.5± 0.21**	9. 5 ± 0.24*	2.7± 0.14**
**Contralateral ovary moclobemide (10 mg/kg)**	10	4.3± 0.16	8.9± 0.26	2.9± 0.22
**Contralateral ovary moclobemide(20 mg/kg)**	10	4.2± 0.18	8.6± 0.29	3 ± 0.25
**Intact control**	10	4.4± 0.24**	8.5± 0.3**	3.1± 0.34**
**I/R control**	10	10.4 ± 0.24	17.4 ± 1.11	1.03 ± 0.11
**Contralateral ovary I/R**	10	6.5± 0.38	11.7 ± 0.54	1.9± 0.19


Results were the mean ± standard deviation. ** Refers to p<0.0001, * refers to p<0.001 when compared to I/R control group.

## Discussion

This study investigated the levels of MDA, NOx, and GSH in rat ovarian tissue and contralateral ovarian tissue subjected to 3 hours ischemia and 2 hours reperfusion injury followed by the histological evaluation of those tissues. These taking steps were appropriate in inducing damage in ovaries (23). The results showed that moclobemide, at doses of 10 and 20 mg/kg, significantly limited the increase of MDA and NOx levels, also it significantly increased GSH level compared to the control group following the application of I/R. All doses of moclobemide (10 and 20 mg/kg) decreased the level of NOx when compared to IR control group. Contralateral ovarian tissue of IR control group showed a mild increase and decrease in antioxidant levels observed in oxidantive parameters. In the contralateral ovarian tissue of 10 and 20 mg/kg moclobemide groups, the levels of MDA and NO were found lower in comparison to IR group. Histological examination showed mild edema and vascular congestion in the above-mentioned groups, as compared with a moderate grade of both at the lower dose. The ovaries of the control group exhibited much more serious edema and vascular congestion than did in either of the treatment groups. Haemorrhage and leukocyte infiltrates were also observed in the control group after I/R. In the contralateral ovarian tissue of IR control group, mild edema and vascular congestion was observed. This demonstrates a correspondence of the histological findings with the biochemical analysis results. The level of MDA (the end product of lipid peroxidation), which we measured in the ovarian tissue, was found to be higher following injury compared to the healthy ovarian tissue (24, 25). MDA leads to cellular damage by causing polymerization and cross-linking of membrane components (26), also is generally used as an index for oxidative stress (27).

NOx production decreased in the ovarian tissue of both moclobemide groups in comparison to the control group after I/R. Endothelial-derived nitric oxide (EDNO) or endothelium-derived relaxing factor (EDRF) may be released into the circulation system in cases of hypoxia, endotoxin discharge, or stress from cellular trauma (8). Reperfusion injury is created through a complex mechanism involving endothelial factors (NO) and WBC in addition to ROS. We reasonably speculated that the event triggered the injury was the damage to the endothelial cells (3, 4). NO is synthesized from nitric oxide synthase enzyme. There are usually three isoforms of nitric oxide synthase (NOS): endothelial NOS (eNOS), inducible NOS (iNOS), and neuronal NOS (nNOS). In the ovary, there are two types of NOS isoforms (iNOS and eNOS), but expression and activity of iNOs and eNOS greatly vary in different animal species and ovarian processes. The studies have showed that the changed expression of iNOS during ovulation process is not clear, and the expression and functional activity of nNOS during follicular development and ovulation are not demonstrated in any mammalian species (28, 29). However, eNOS have demonstrated as the dominant isoform of NOS in ovarian tissue (30).

The excessive rise in the intracellular NO concentration initiates the toxic events that lead to cell death (31). The release of oxygen is parallel to that of NO, and they influence each other to produce hydroxyl radical (OH) and nitrogen dioxide (NO_2_). During this reaction, toxic intermediates like peroxynitrite and peroxynitrous acid arise (32). As a result, NO is indicated as a mediator of the reperfusion phase of I/R.

Cells are not resistant enough to injury by ROS. The harmful effects of the free radicals are not generally seen because of the antioxidant defence mechanisms developed against such effects (33). However, oxidative injury can develop when the created imbalance is in favour of oxidation (26). Besides the many endogenous mechanisms inhibiting I/R injury, there are numerous exogenous drugs that can limit such injury (26, 34). Oxidative stress affects ovarian tissue as well as uterine tissue (35). In our study, moclobemide was shown to be one of the exogenous substances that can limit I/R injury in ovarian tissue. The GSH level in the ovarian tissues of the animals treated with moclobemide was found to be significantly higher than in the control tissues after I/R injury.

GSH is the most important antioxidant compound. It is synthesized in many tissues from glutamate, cysteine, and glycine. GSH protects the sulphydryl (SH) groups in proteins against oxidation by keeping them in a reduced state, thus preventes the inactivation of functional proteins and enzymes. GSH also plays a role in the detoxification of alien compounds and the membrane transport of amino acids (36). An experimental work has showed that GSH deficiency causes oxidative stress and potentially results the various diseases (37). As mentioned above, reperfusion damage occurs through a complex mechanism involving ROS, EDNO, and neutrophils (3, 4). As a result, antioxidant treatment is a necessity during I/R until surgical correction will be performed. Some publications have supported our idea of antioxidant administation by indicating that antioxidant treatment prevents or delays disease if oxidative injury plays a role in its origin or development (38, 39).

## Conclusion

While the severe oxidative damage was detected and pathological results showed that in the ovarian tissue of IR control group, the contralateral ovaries showed mild result. In 10 and 20 mg/kg doses, moclobemide repressed oxidative damage in I/R damage, significantly; however, in the contralateral ovaries, it inhibited oxidative damage, totally.
